# Editorial: Development of Student Understanding: Focus on Science Education

**DOI:** 10.3389/fpsyg.2019.02861

**Published:** 2019-12-18

**Authors:** Calvin S. Kalman, Mark Lattery

**Affiliations:** ^1^Department of Physics, Concordia University, Montreal, QC, Canada; ^2^Department of Physics, University of Wisconsin–Oshkosh, Oshkosh, WI, United States

**Keywords:** conceptual change, knowledge in pieces, cognitive dissonance, epistemological beliefs, critical thinking

How can we engage a broad audience of science education researchers and practitioners to examine strategies to help students become more expert-like in their thinking? To succeed in a technologically evolving society, students must engage in critical thinking, collaborative problem solving, and evidence-based reasoning. What specific kinds of interventions are needed to assist students with varying epistemologies to attain these skills?

Many students see scientific knowledge as unconnected and conveyed by authorities, such as the instructor and the textbook; correspondingly, their own knowledge structure is fragmented and disordered—a “knowledge in pieces” (KIP) as diSessa (1983). However, many other students enter the classroom with semi-coherent and relatively stable alternative conceptions about how the world works, and also an instinct for the nature of science or scientific knowledge; e.g., students “are authentic and creative scientific modelers” (Lattery, 2017, p. 109). Whether student scientific knowledge is best characterized as a fragmented or coherent, the instructor is confronted with the difficult task of bridging student's prior knowledge with target ideas. The task is especially challenging if the student's ideas are profoundly different (“incommensurable”) with target ideas. Chi (2013) noted that many concepts in student's initial flawed mental models are not transformed to the accepted scientific model despite repeated corrections or patchings of the underlying rules.

We launched this ebook to consider instructional supports that are necessary for students to examine and develop their own ideas and compare them to the ideas presented by peers, the textbook, and the instructor. This is a follow up to our previous review of three instructional strategies that show promise to address this challenge in the context of an introductory physics classroom (Kalman and Lattery, 2018). More details are also found in Kalman (2017).

In this Research Topic, ten articles touch on various aspects of helping students become more expert-like in their thinking. Four articles were submitted through *Frontiers in Education STEM Education* and six articles through *Frontiers in Psychology Educational Psychology*.

In her article, Vosniadou directly addresses the structure of students' knowledge. She cites arguments in the research literature that children start the knowledge acquisition process by forming beliefs based on their everyday experiences and lay culture. In her view “the development of science knowledge is a long and gradual process during which students use constructive learning mechanisms to assimilate new, scientific, information into their prior knowledge causing hybrid conceptions—or misconceptions. Science instruction needs to help students become aware of their experience-based beliefs that might constrain science learning causing misconceptions, provide information gradually based on students' learning progressions and develop students' scientific reasoning and executive function skills.”

Hadzigeorgiou and Schulz's article is part of an extended research project investigating how to improve secondary students' motivation and engagement to learn about science. This article focuses on students' “narrative mode of thought” as a bridge to understanding science.

Seufert notes that learning with text and pictures requires learners to integrate the given information into one coherent mental representation. “Since learners often fail to integrate text and pictures, the study investigates the effects of a training for text processing strategies, picture processing strategies and strategies to map text and picture onto each other.”

Kerwer and Rosman examines the dependence of epistemological change on the (un)resolvability of contradictory information, the extent to which explicit reflection on diverging information supports epistemic change, and how topic-specific diverging information affects topic- and domain-specific epistemic beliefs.

Zhao et al. show that information that displays more concrete characteristics exerted a greater cognitive inhibitory effect during the working memory task, and a greater cognitive inhibitory effect was produced when all of inhibition retrieval information clues are provided than when none of the clues are provided in the working memory task.”

Kaiser and Mayer investigate the benefits of combining example-based learning with physical, hands-on investigations in inquiry-based learning for acquiring scientific reasoning skills.

Four papers concentrate on students' conceptual understanding. Nunez-Oviedo and Clement focus is on how whole class discussions can contribute to the learning of conceptual models in science. As they point out, “Science educators today still struggle with finding better ways to help students develop strong conceptual understandings as opposed to memorizing isolated facts.” “It is possible to start from student-generated models that conflict with the target model in a number of ways, and still arrive at the target model for the lesson through discussion.”

Han and Ellis describe how the phenomenographic method can be used to develop students' conceptual understanding of scientific concepts, to inform effective instructional design in science teaching, and to identify and improve evidence-based factors in student learning to enhance learning outcomes in science.

Munoz-Rubke et al. consider how learning formal concepts becomes more meaningful when teachers integrate what children already know and also underscore that spatial abilities have a strong and positive effect both on the motivation to learn math and on math performance itself.

Bigozzi et al. use a semi-structured interview to question faculty about their ideal teaching approach and their actual teaching approach. They also examined which component of the teaching approach is associated with students' progress in physics and critical thinking skills. The authors note that “simply going to the laboratory does not foster a constructivist learning in students, unless it is matched with reflection.”

This collection of papers will hopefully engage a broad audience to extend the results presented by the authors of the articles found in this ebook to find additional ways to help students become more expert-like in their thinking.

## Author Contributions

All authors listed have made a substantial, direct and intellectual contribution to the work, and approved it for publication.

### Conflict of Interest

The authors declare that the research was conducted in the absence of any commercial or financial relationships that could be construed as a potential conflict of interest.
